# Assessing dairy calf response to long-distance transportation using conditioned place aversion

**DOI:** 10.3168/jdsc.2022-0209

**Published:** 2022-04-26

**Authors:** Katherine C. Creutzinger, Kendra Broadfoot, Hanne M. Goetz, Kathryn L. Proudfoot, Joao H.C. Costa, Rebecca K. Meagher, David L. Renaud

**Affiliations:** 1 Department of Animal and Food Science, University of Wisconsin-River Falls, River Falls 54022; 2 Department of Population Medicine, University of Guelph, Guelph, ON, Canada N1G 2W1; 3 Departments of Health Management and Companion Animals, Atlantic Veterinary College, University of Prince Edward Island, Charlottetown, PEI, Canada C1A 4P3; 4 Department of Animal and Food Sciences, University of Kentucky, Lexington 40506; 5 Department of Animal Science and Aquaculture, Dalhousie University, Truro, NS, Canada B3H 4R2

## Abstract

Surplus neonatal dairy calves are routinely transported long distances from the dairy farm of birth to calf raisers. Most of the research assessing the effect of transportation on young calves has focused on physiology and health, without considering calf affective states. The aim of this study was to assess the affective response of young male dairy calves to transportation using conditioned place aversion (CPA) testing. Three bouts of transportation exposed dairy calves (n = 95) to transportation for 6 (n = 31), 12 (n = 32), or 16 h (n = 30). During transportation, the trailer interior was made visually distinct using blue horizontal (n = 2 groups) or red vertical (n = 1 group) stripes. Distinct color and pattern combinations were used to create a visual association with transportation. A subset of calves (6 h = 14; 12 h = 14; 16 h = 14) were selected for CPA testing if they were ≥7 and <28 d of age at the time of testing after transport. A group of sham calves that were not transported (n = 13) and were ≥7 and <28 d of age at the time of testing also were enrolled. Wall coverings with the same color and pattern of those in the trailer were fixed in individual sham calf stalls for 6 h on the morning of transportation. Before transportation, calves were placed in a 3-compartment test apparatus with neutral walls and were able to move about freely for 15 min to habituate them to the test apparatus. Calves were CPA tested on d 2 after transportation. During the posttest, calves were placed in the test apparatus with 3 compartments including a neutral center pen (center), blue horizontal stripe compartment (side), and red vertical stripe compartment (side) for 30 min; calves were able to move throughout the apparatus freely. Time spent in each compartment was recorded using continuous observation and analyzed as a percentage of total time in the apparatus. Calves spent less time in the compartment associated with transportation compared with the compartment with no prior association (22% vs. 42% ± 3% of time in the transportation-association and no-association compartments, respectively); however, this behavior was not affected by transportation treatment. Results from this study suggest that calves may perceive transportation to be aversive regardless of duration but may also be attracted to exploring a novel stimulus.

## Highlights

• Calf affective states need to be taken into consideration when assessing the effect of long-distance transportation on welfare.
• Following conditioning, calves spent more time in a test apparatus section with no prior association than a section conditioned to transportation or a home pen.
• Calves may have spent more time in the compartment with no prior association because of novelty.
• Novelty seeking is an important confounder to consider when using condition place aversion testing.

## Graphical Abstract

**Figure:**
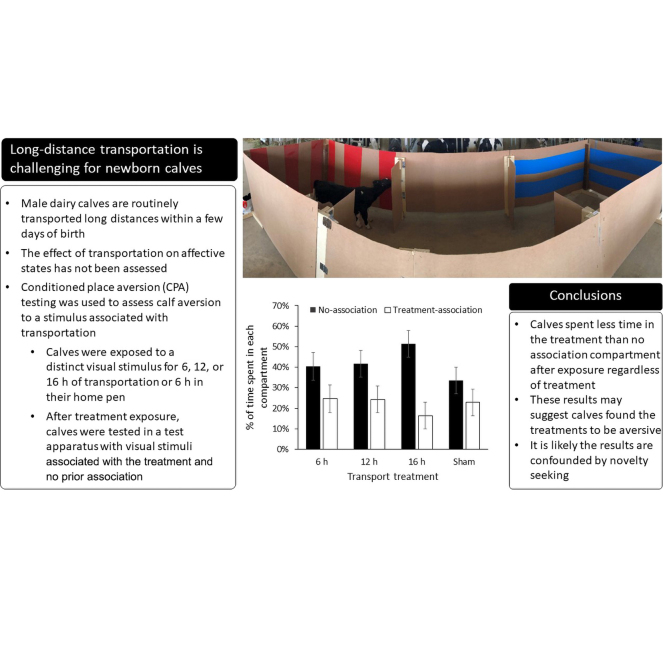
**Summary:** Surplus dairy calves are routinely transported long distances shortly after birth to calf raisers. This study aimed to assess the effect of long-distance transportation on calf affective states by conditioning calves to a visual stimulus associated with the interior of a trailer or the interior of their individual stall if they were transported or not, respectively. After treatment exposure, calves were tested in a 3-compartment apparatus containing a novel and treatment associated stimulus, and neutral center compartment. Calves spent less time in the treatment than novel stimulus compartment regardless of treatment. However, several methodological challenges may have affected the results.

Young dairy calves are routinely transported from the dairy farm on which they are born to a calf-raising facility during the first week of life (Shivley et al., 2019). Despite being a common practice, long-distance transportation can have negative implications for calf welfare. Calves typically do not have access to food or water during transportation, likely resulting in hunger and dehydration (reviewed by Roadknight et al., 2021). Social stressors are also common during transportation, including mixing with unfamiliar animals and exposure to novel environments (reviewed by Creutzinger et al., 2021). To date, most of the research assessing the effect of transportation on young calves has focused on short-term physiological changes and disease after transportation with little investigation into in affective state responses (Goetz et al., 2022). To fully understand the effect of transportation on calf welfare it is important to assess calf emotional and mental states (i.e., affective state) in response to transportation.

Conditioning paradigms, such as classical or respondent conditioning, can be used to assess an animal's motivation to avoid or experience a stimulus (Prus et al., 2009). Measuring the amount of time an animal spends in an area that has been associated with a stimulus can indicate their like or dislike for the stimulus. For example, rats prefer areas associated with opiates (Bozarth, 1987) and psychostimulants, such as amphetamine and cocaine (Khroyan et al., 1999). Conditioned place avoidance or preference (**CPA/CPP**) tests are most commonly used in drug trials but have also been used for stimuli including food (Velázquez-Sánchez et al., 2015), social interaction (Calcagnetti and Schechter, 1992), and copulation (Jenkins and Becker, 2003). Recently, CPA tests have been used as a measure of avoidance in young dairy calves, where Ede et al. (2019) found that dairy calves avoided visual stimulus associated with disbudding, which is a painful experience. No research to our knowledge has used CPA to assess the effect of transportation on farm animals. The objective of this study was to use CPA testing associated with long-distance transportation by road to assess young male dairy calf aversion to transportation. We predicted duration of transportation would increase the aversiveness of the experience and therefore calves' avoidance of the testing apparatus compartment associated with transportation.

This project was part of a larger study to assess the effect of transportation on physiological and health outcomes of young dairy calves [H. M. Goetz, K. C. Creutzinger, D. Kelton (Department of Population Medicine, University of Guelph, Guelph, ON, Canada), J. H. C. Costa, C. Winder (Department of Population Medicine, University of Guelph, Guelph, ON, Canada), and D. L. Renaud; manuscript in under preparation]. Animal use was approved by the University of Guelph Animal Care Committee (Animal Use Protocol #4430). A total of 3 transportation bouts from April 2021 to June 2021 were carried out in southern Ontario, Canada. Male and female surplus calves (n = 97) were enrolled in this study on the day of birth from 5 dairy farms in southern Ontario, within 50 km of the University of Guelph. Holstein (n = 52) and dairy-beef crossbred calves (n = 45) were included in the study. On the day of transportation, calves were randomly assigned to 1 of 3 treatments: (1) 6 h (n = 31), (2) 12 h (n = 32), and (3) 16 h (n = 30) of continuous transportation by road. Calves were 9 ± 5 d of age (range: 1 to 18 d) on the day of transportation.

On the dairy farm of birth, calves were housed indoors in individual pens bedded with sawdust or straw. The calves were fed and managed according to producer protocols developed in conjunction with their herd veterinarian. After transportation, calves were individually housed in hutches outdoors; additional space outside of the hutch was not provided. Straw was used as bedding during cold months (October to April) and sawdust was used as bedding during warm months (May to June). During the first week after arrival, calves were provided 6 L of milk replacer (150 g/L) per day, fed twice per day (0800 and 1800 h) in 3-L increments using a nipple bottle. Water and solid feed were available ad libitum starting d 2 after arrival to the calf-raiser.

A subset of calves from the trial were selected for the CPA study. Fifty-five male and female (male = 42, female = 13) Holstein (n = 39) and dairy-beef cross calves (n = 16) were enrolled in the CPA test at 10 ± 6 d of age (mean ± SD); age was similar between treatment groups (*P* = 0.78). Calves were eligible for the CPA study if they were ≥7 and <28 d of age at the time of the testing after transportation (posttest). Specifically, 42 male calves from the larger study were selected for CPA analysis (6 h, n = 14; 12 h, n = 14; 16 h, n = 14), whereas an additional group of female calves (n = 13) was selected to serve as a sham treatment group. Female calves were chosen as the sham group of calves because they were replacement heifers for the dairy farm and were not going to be transported. All sham calves were Holstein. If dairy-beef and male Holstein calves were eligible for CPA testing (i.e., ≥7 d of age at testing), they were old enough to be transported and were removed from the farm at the time of transportation; thus, all dairy-beef and male Holstein calves on the dairy farms were transported for the study. An attempt was made to balance breed between the transported groups (6 h: Holstein = 9, dairy-beef = 4; 12 h: Holstein = 7, dairy-beef = 7; 16 h: Holstein = 9; dairy-beef = 5; sham: Holstein = 13, dairy-beef = 0). Calves were enrolled from 3 study dairy farms; 3 out of the 5 study farms were used because they had sufficient space to set up the test apparatus indoors.

The test apparatus was constructed from plywood (4.88 m long × 1.83 m wide × 1.22 m high) and separated into 3 compartments (1.83 m long × 1.83 m wide × 1.22 m high) using half walls within the pen (0.46 m long). At the time of testing before transportation (pretest), the interior of the pen was unaltered plywood with which calves had no prior association. No colors were visible at the time of the pretest so that calves would not form an association with the transportation visual stimuli before transportation. At the time of the posttest, the pen contained visually different stimuli in each compartment: red vertical stripes (side), neutral unaltered plywood (center), and blue horizontal stripes (side). The color and pattern combinations were chosen based on previous research that found young dairy calves differentiated between red squares and blue triangles (Ede et al., 2020, 2019) and a solid black wall and a white wall with black stripes (Adcock and Tucker, 2020). The color in the test apparatus not associated with transportation was novel to all calves at the time of the test. The sham group of calves were not transported, but paper with the same color and pattern that was in the trailer was attached to the interior of the individual stall on the same morning calves were transported and remained in place for 6 h before removal.

This CPA study used an unforced choice test (i.e., 3 compartments with a neutral choice did not require animals to choose between side compartments) and an unbiased design (i.e., the side associated with stimulus is determined by the researcher, regardless of preference of each subject for either compartment before conditioning; Prus et al., 2009). Two days before transportation, calves were exposed to a pretest. They were placed in the same test apparatus for 15 min with free access to all compartments of the apparatus with no color scheme present. Calves that failed to explore all compartments of the apparatus were excluded (6 h, n = 1).

On the day of transportation, calves were loaded into a gooseneck trailer (9.1 m × 2.3 m) deep bedded with chopped straw (approximately 15 cm deep). Space allowance inside the trailer was 0.71 ± 0.15 m^2^ per calf (mean ± SD). The truck and trailer drove continuously for 16 h, stopping at 6, 12, and 16 h to unload calves at a calf-raiser in southern Ontario according to their assigned treatment group. Brown builder's paper (0.91 m; HDG Brown Builders Paper; Trimaco) was attached to the inside of the trailer using tape (Figure 1). The paper was painted with either red vertical or blue horizontal stripes before installation in the trailer. Blue horizontal stripes were installed inside the trailer on the first group of transported calves, then alternated between red vertical and blue horizontal stripes, resulting in 2 blue horizontal stripe groups (n = 19) and 1 red vertical stripe group (n = 33). Two calves (6 h, n = 1; 12 h, n = 1) were excluded from the CPA test because they were deemed to be unfit for transportation and remained on the dairy farm of birth.

**Figure 1:**
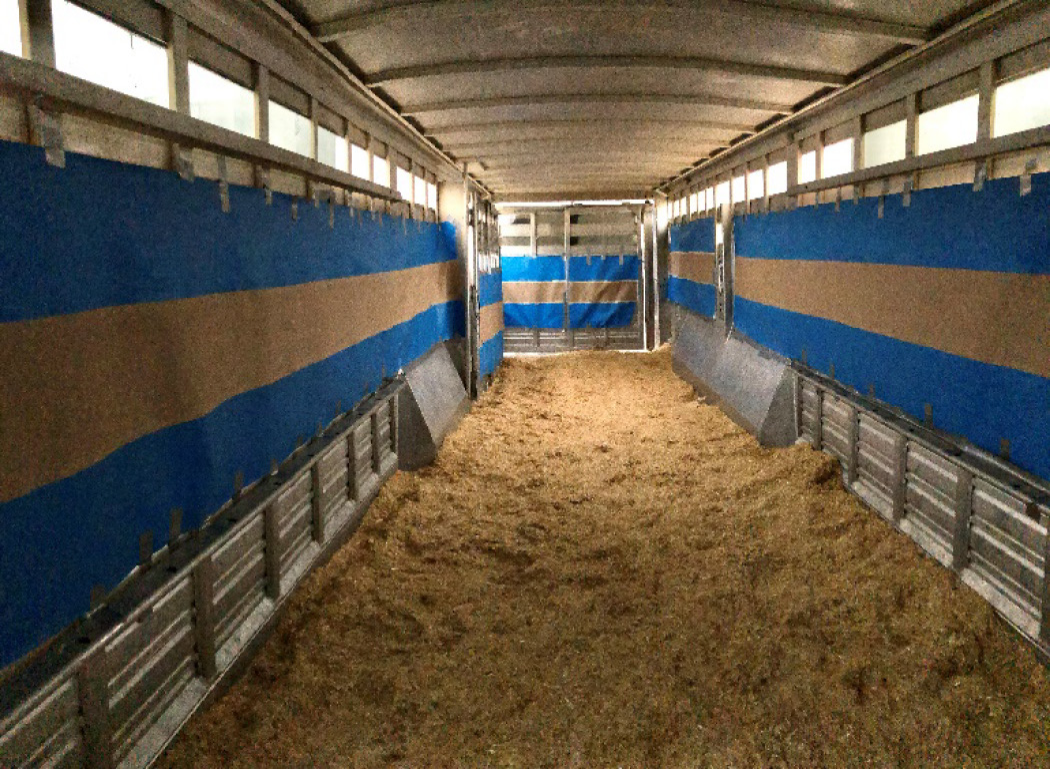
Painted paper (blue horizontal or red vertical stripes) was installed in a gooseneck trailer in which Holstein and dairy-beef calves were transported for 6 h (n = 12), 12 h (n = 13), or 16 h (n = 14) to condition calves to a visually distinct stimuli associated with transportation.

The posttest was performed 2 d (n = 39; 6 h = 10, 12 h = 9, 16 h = 8, sham = 13) and 3 d (n = 13; 6 h = 3, 12 h = 4, 16 h = 6, sham = 0) after transportation due to time constraints. Sham calves that were not transported were tested at the dairy farm of birth and calves that were transported were tested at the calf-raiser to which they had been transported. Testing for all calves occurred after the morning feeding, beginning at approximately 0800 h. Sham calves were tested within 1 h before transported calves in group 1 and were tested congruent to transported calves for groups 2 and 3. The test apparatus was assembled inside a well-lit barn. Each calf was placed in the test apparatus for 30 min. Two handlers moved calves from individual housing to the test apparatus using low stress handling methods and a halter as needed. Care was taken by the test administrators that the calves were undisturbed by human interference while in the test apparatus.

The CPA tests were continuously recorded using a GoPro HERO7 and GoPro HERO8 (GoPro Inc.). Cameras were mounted above the test apparatus so that the calf was always visible in all areas of the apparatus. Videos were recorded and saved on MicroSD cards (Ultra MicroSDXC; SanDisk LLC) until later analysis. Videos were reviewed by a single observer in a VLC media player (VideoLAN) using continuous observation. The time at which calves entered a new compartment was recorded. Calves were defined as entering a new compartment when both front legs were in the compartment (Ede et al., 2020). The number of times calves entered a new compartment in the apparatus was recorded as a measure of activity during the posttest. The amount of time (min) spent in each compartment and the total number of movements between compartments was summarized per calf.

A sample size of 12 animals per treatment was calculated for a statistical power of 0.8 and significance level of 0.05. Mean difference and standard deviation values were based on results from Ede et al. (2019). All statistical analyses were performed with SAS software (version 9.4, SAS Institute Inc.). Raw data were visually screened for data distribution and outliers using the UNIVARIATE procedure in SAS. Data were normally distributed, and no outliers were detected. Statistical significance was declared at *P* < 0.05, and tendencies at *P* < 0.10. The normality of residuals was visually assessed using residual plots.

Time spent in the central compartment of the apparatus was not analyzed because calves had to cross through it to reach both the left and right compartments, which could have inflated the amount of time calves spent in the center compartment. Hence, the amount of time in each compartment was assessed as a percentage of total time in the test apparatus. Univariable analysis was conducted between dependent variables, and explanatory variables (i.e., age, breed, day of CPA posttest) were analyzed using linear regression models (PROC MIXED), and variables with *P* < 0.20 were included in the final model. Sex was not included as an explanatory variable to avoid collinearity with the dependent variables. No explanatory variables were associated (*P* < 0.20) with the dependent variables; thus, they were not included in the multivariable models. Percentage of time in each compartment was analyzed using linear mixed-effect models (PROC MIXED); compartment (transport-association color compartment vs. no-association color compartment), treatment, and a compartment × treatment interaction were included as fixed effects, and transportation group was included as a random effect. Post hoc analysis was used to determine treatment differences using the LSMESTIMATE statement; a Bonferroni adjustment was applied.

The total number of times calves entered a new compartment (i.e., activity) was also analyzed using a linear mixed effects model (PROC MIXED). Treatment was included as a fixed effect and transportation group as a random effect. Post hoc analysis was used to determine treatment differences using the LSMESTIMATE statement. Specifically, the sham group was compared with all transportation groups based on initial analysis. A Bonferroni adjustment was applied.

A total of 52 calves were included in the CPA posttest (6 h = 12, 12 h = 13, 16 h = 14, sham = 13). After transportation, calves spent less time in the compartment associated with transportation than the compartment with no prior association, regardless of treatment (LSM ± SE: 22% vs. 42% ± 3% of time in transport-association vs. no-association compartments; *P* < 0.001). There was no effect of treatment on the amount of time spent in each compartment (*P* = 0.83) and no interaction was found between compartment and treatment (transport-association vs. no-association: 6 h = 25% vs. 40%; 12 h = 24% vs. 42%; 16 h = 16% vs. 51%; sham = 23% vs. 34%; SE = 7%; transport-association: *P* = 0.27; Figure 2). It is not clear whether calves, including those in the sham treatment, avoided the transportation-associated compartment because they had a negative association with that stimulus or if they preferred the no-association color because it was novel. Novelty seeking is considered a main limitation of CPA/CPP tests used in rodent trials (Prus et al., 2009). To avoid novelty seeking in rodent trials, it is recommended that animals first have free access to the entire testing apparatus for several days (e.g., 3 to 5 d). Repeated exposure to all environments allows animals to habituate to the testing apparatus, which eliminates novelty as a confounding variable (Bardo and Bevins, 2000). Ideally, calves would have been conditioned to transportation in the apparatus and the other stimulus (i.e., opposite color) would be conditioned to a neutral stimulus (e.g., on the trailer without transportation). Conditioning would then occur multiple times in each compartment over multiple days (Prus et al., 2009). In the case of this study, repeated conditioning to long-distance transportation and a neutral stimulus in the apparatus was not practical or humane, and thus was not possible to achieve in this study. As novelty seeking may be a major limitation of our study, we encourage future research to follow-up with this work using methods to reduce novelty seeking.

**Figure 2:**
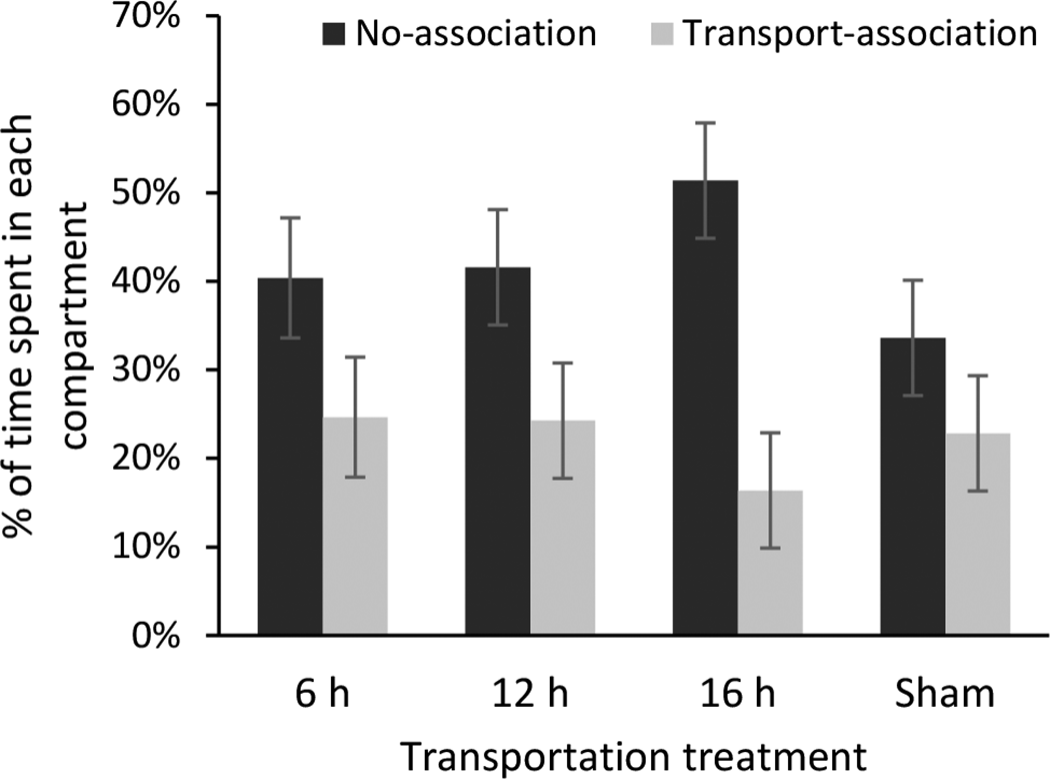
The percentage of time calves spent in each compartment in relation to transportation treatment (LSM ± SE). Treatments included 6-, 12-, and 16-h continuous transportation by road, and sham (not transported). Transportation color association was formed by installing painted paper in the trailer during transportation or the sham calf individual stall for 6 h.

Calves in the transport and sham groups had different experiences that may have affected their behavior. For example, transported calves were moved to a new location after transportation, which was accompanied by changes in feed, feed availability, and housing. By comparison, sham calves remained on the farm of birth in a familiar environment with consistent feed and feed availability. These differences may have affected the calves' response to novelty. For example, previous research has demonstrated that calves housed individually in barren environments are more anxious when exposed to novelty than those housed socially in enriched environments (Jensen et al., 1997). It is possible that in this study nontransported (sham) calves were more likely to seek novelty than transported calves because their overall experience during the study period was less negative than transported calves and they had experienced fewer changes to their environment.

Differences in behavior may have also been affected by calf sex, as all sham calves were female and all transported calves were male. Calf sex may have affected the novelty seeking response; Wagner et al. (2013) found that preweaning female dairy calves explored a test arena less than males in an isolation test. In the future, we recommend balancing for sex across treatment groups when attempting a CPA test.

Both transported and nontransported calves spent less time in the apparatus compartment to which they were conditioned. Again, these calves may have been seeking novelty. Alternatively, sham calves kept on farm in individual pens may have also made a negative association with the visual stimuli conditioned to their home pen. Indeed, research has found that individually housed calves respond more pessimistically to ambiguous stimuli in a reward or nonreward cognitive bias test than pair-housed calves, suggesting that individual housing has negative effects on calf affective states (Bučková et al., 2019). Thus, it is possible that calves in this study avoided the conditioned stimuli if they perceived being housed individually negatively. To avoid conditioning with an environment that calves have experienced, future research should choose a sham treatment that provides exposure to the treatment environment without the actual exposure effects (i.e., condition in the apparatus in a stationary trailer for a short period of time).

Transported calves spent less time in the compartment conditioned to transportation, which is consistent with previous studies that found calves spent less time in test apparatus compartments conditioned to disbudding pain (Ede et al., 2020, 2019). This finding may reflect transported calves' aversion to the transportation-associated stimulus, although more research is encouraged to replicate this finding with fewer methodological challenges. We hypothesized that longer durations of transportation would be more aversive to calves; however, we did not find differences between calves transported for different lengths of time. A lack of treatment differences may suggest that transportation was aversive to all calves, including those transported for 6 h.

Interestingly, the number of movements between compartments during the posttest was affected by treatment (*P* = 0.03). Calves that were not transported had a greater number of movements between the compartments than all transported calf treatments (i.e., were more active in the test apparatus; Figure 3). Calves housed individually are more active in novel test arenas than calves housed in enriched environments with social partners (De Paula Vieira et al., 2012), suggesting that the test arena may have been rewarding due to the greater space allowing for increased locomotor play. In our study, the home pens of sham and transported calves were less than half the size of the testing apparatus, perhaps making access to the large area valuable for the calves (Jensen, 2001; Sisto and Friend, 2001). Anecdotally, we observed running and locomotor play behavior in calves, especially during the pretest. However, we did not record these responses because they were outside the scope of CPA testing. Alternatively, the difference in movement may be due to calf recovery from transport. Preliminary data from this study (Creutzinger et al., 2021) found that calves transported for 12 and 16 h spent more time lying than calves transported for 6 h for up to 4 d after transportation. Less activity in the test apparatus for transported compared with nontransported calves may be indicative of transported calves having less energy after transportation. There are multiple possible explanations for differences in activity between transported and nontransported calves but without greater environmental control or clarifying experiments it is difficult to interpret the results from this study.

**Figure 3:**
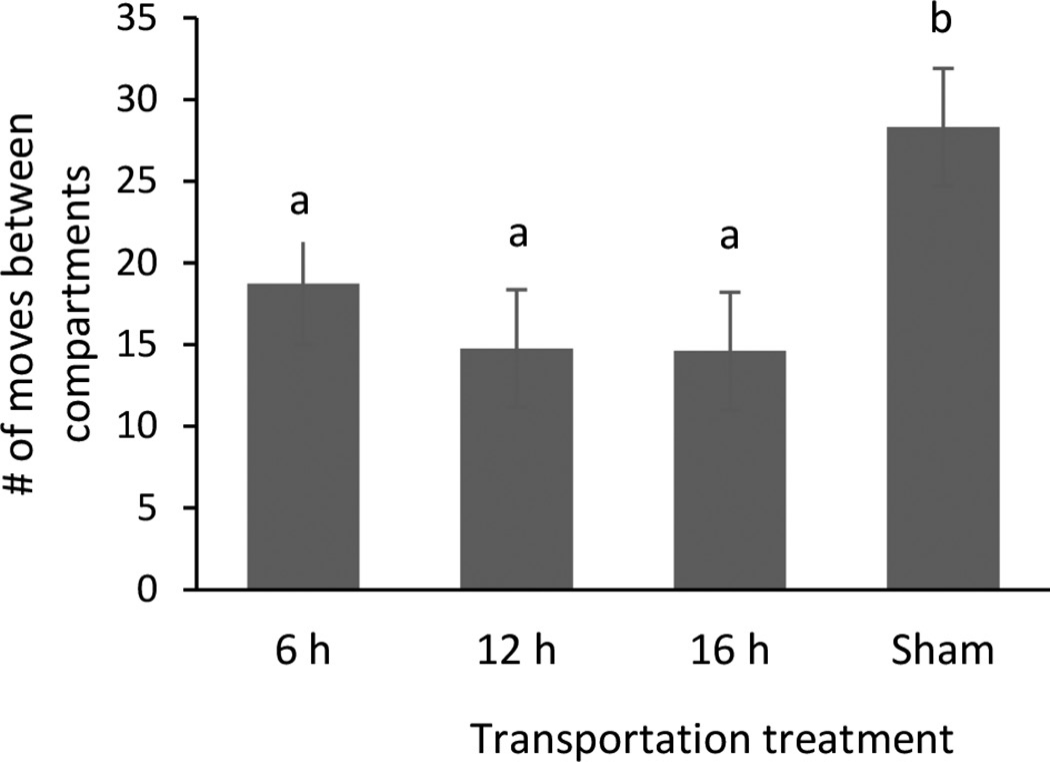
The number of times calves moved between compartments during the posttest in relation to treatment (LSM ± SE). Treatments included 6-, 12-, and 16-h continuous transportation by road, and sham (not transported). Different letters (a, b) indicate a significant difference (*P* < 0.05).

This was the first study to our knowledge to explore if young dairy calves perceive transportation to be aversive. Calves spent less time in the transportation-associated compartment than the compartment with no prior association regardless of transportation duration, which suggests that calves found all lengths of transportation to be aversive. These results, however, must be taken with caution as they may be confounded with novelty seeking and other methodological challenges. We encourage future research to investigate other tools to assess calf affective states in response to transportation.
